# Relationship of weight-adjusted waist index and body mass index with infertility: A NHANES 2015 to 2018 cross-sectional study

**DOI:** 10.1097/MD.0000000000050059

**Published:** 2026-08-07

**Authors:** Siqi Zhang, Baoyi Liao, Yulu Zhou, Yixuan He, Junjie Cao, Xiaoming Huang

**Affiliations:** a School of Clinical Medicine, North Sichuan Medical College, Nanchong, Sichuan, China; b Department of Clinical Nutrition for Consultation, Beijing Anzhen Nanchong Hospital of Capital Medical University & Nanchong Central Hospital, Nanchong, Sichuan, China.

**Keywords:** body mass index, infertility, NHANES, obesity, weight-adjusted-waist index

## Abstract

Obesity is a significant risk factor for female infertility, representing a growing global health concern. Body mass index (BMI) has traditionally been used as the standard measure of obesity; however, the weight-adjusted waist index (WWI) has recently appeared as a novel metric that may provide a more accurate assessment of obesity. This study aims to investigate the association of both BMI and WWI with female infertility. The “2015–2018 National Health and Nutrition Examination Survey” (NHANES) was utilized to derive the data. The study included 2393 women between the ages of 20 and 50 years. Among them, 251 were diagnosed with infertility. Multivariate logistic regression, subgroup analysis, interaction testing, smooth curve fitting, and receiver operating characteristic curve analysis were conducted to examine the associations of WWI and BMI with infertility. Among women, the infertility’s prevalence was positively associated with both WWI and BMI levels. Higher WWI was significantly related with raised infertility prevalence (OR = 3.63, 95% CI: 1.96–6.73), as was higher BMI (OR = 2.27, 95% CI: 1.29–4.01). In the WWI subgroup analysis, the association was consistently observed in most subgroups, except for age, race, and education level. In the BMI subgroup analysis, the association was consistent across all subgroups. In the population-based cross-sectional data of this study, WWI and BMI were positively associated with the odds of infertility among US women. This finding suggests that obesity-related measures may have potential value in clinical practice and may help to identify potentially high-risk groups of women with infertility. In addition, the existing results suggest that weight management-related interventions may have potential effects on fertility outcomes, but whether they can reduce the infertility rate, explain the biological mechanism, and guide clinical decision-making needs to be verified by prospective, mechanistic, and interventional trials in the future.

## 1. Introduction

Infertility is medically defined as not achieving pregnancy following at least 12 months of consistent, unprotected intercourse.^[1]^ In the US, about 12.7% of women in their reproductive years pursue annual infertility treatment.^[2]^ Globally, it is estimated that 8 to 12% of couples of reproductive age face infertility.^[3]^ Infertility is experienced by 17.8% of people in high-income countries and 16.5% in low-middle-income countries.^[4]^ As a result, infertility has become a major public health concern worldwide, imposing substantial psychological, social, and economic burdens. Ovulatory dysfunction, male-related infertility, and tubal disease are among the most frequent causes. Notably, accumulating evidence suggests that lifestyle- and obesity-related factors (e.g., excess body weight, smoking) also contribute significantly to impaired fertility,^[2]^ currently affecting more than 600 million adults worldwide.^[5]^ Studies have shown that the combined prevalence of overweight/obesity in women of reproductive age is about 32%.^[6]^ Obesity is strongly linked to impaired fertility, with obese women being more prone to experience menstrual irregularities and ovulatory dysfunction.^[7]^

Body mass index (BMI), a traditional indicator for assessing obesity, is widely used in clinical practice and epidemiological research. Studies have shown an association between BMI and infertility: for each 1 kg/m^2^ increase in BMI, the odds ratio (OR) for infertility risk is 1.03, with a 95% confidence interval ranging from 1.02 to 1.05.^[8]^ However, BMI has significant limitations. It only reflects the ratio of a person’s weight to height and cannot indicate fat distribution, meaning it has obvious limitations in reflecting the distribution and quality of adipose tissue and lean tissue.^[9]^ Excess fat in specific areas, such as abdominal fat, is often closely associated with metabolic disorders and endocrine imbalances. These factors are precisely the key elements that influence female reproductive function.^[10]^ To address this, Park et al (2018) proposed the weight-adjusted waist index (WWI, cm/√kg), a novel anthropometric indicator that incorporates waist circumference (WC) and body weight. Unlike BMI, WWI better reflects body fat distribution and is not influenced by muscle mass, offering a potentially more accurate valuation of obesity-related health risks.^[11]^ Studies have confirmed that WWI is positively correlated with the risk of infertility, and WWI is helpful in identifying infertile women and may play a role in reducing the odds of infertility.^[12]^

Therefore, analyzing the link between WWI, BMI, and infertility is essential to further clarify the obesity’s adverse impact on reproductive health. To this end, we utilized data from the “2015–2018 National Health and Nutrition Examination Survey” (NHANES) to examine the link between WWI, BMI, and infertility among women in the US. The following hypothesis was also made: female infertility is positively associated with WWI and BMI levels.

## 2. Methods

### 2.1. Study design

This study was a cross-sectional study based on NHANES data from 2015 to 2018. The NHANES used stratified, multistage cluster sampling. We included women between the ages of 20 and 50 years in the analyses. WC and body weight were measured according to a standardized procedure. The weight-adjusted WC index (WWI) was calculated as WC (cm)/√ body weight (kg), and BMI was used as the control exposure. The primary outcome was infertility, as assessed by the Reproductive Health questionnaire. For comparison purposes, participants were divided into quartiles (Qs) based on the distribution of WWI and BMI. The complex sampling design of the NHANES was considered in the statistical analysis. The NHANES protocol was reviewed and approved by the National Center for Health Statistics Ethics Review Board, and written informed consent was obtained from all participants.

### 2.2. Study population

**Figure 1.**
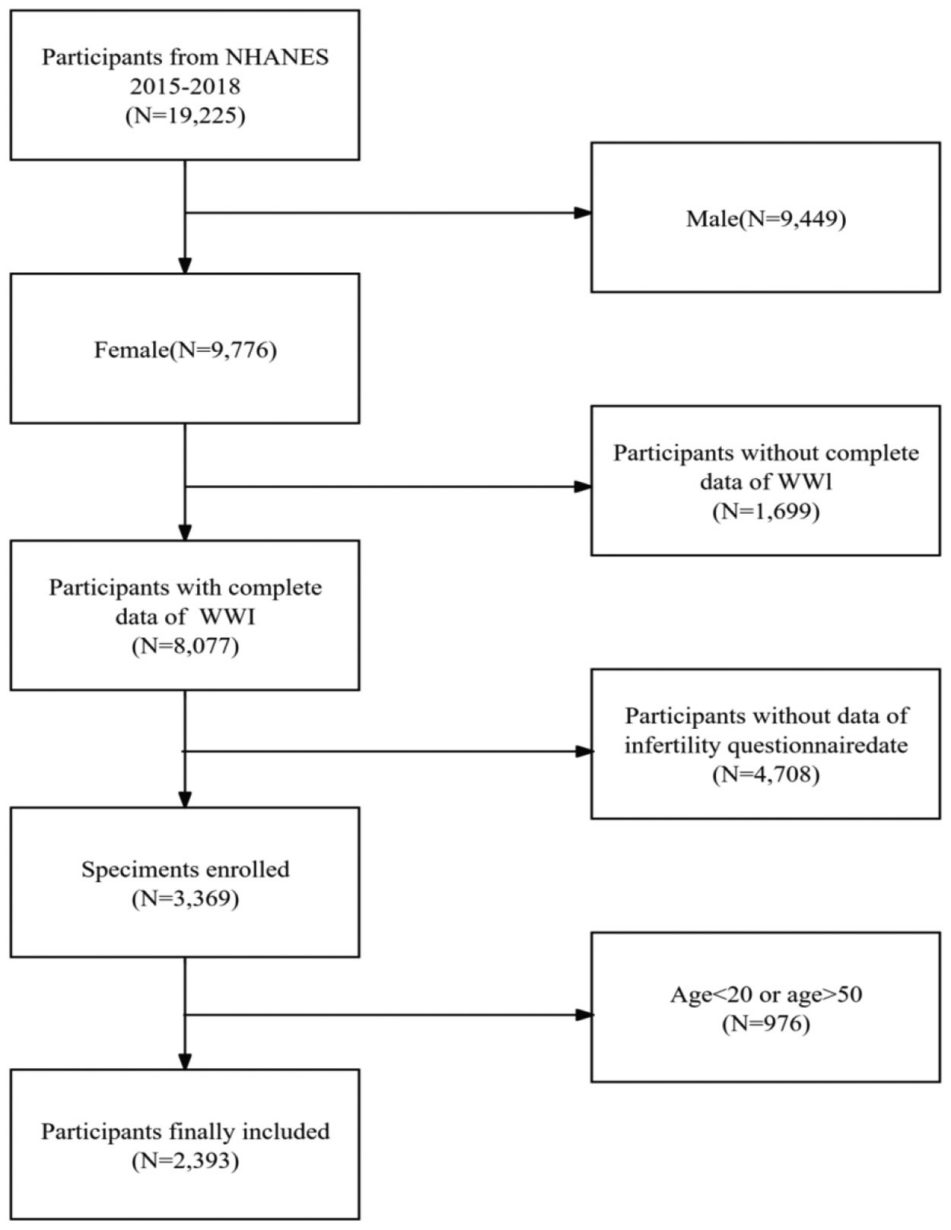
Flow chart of participant selection.

The NHANES is a nationally illustrative survey led by the “National Center for Health Statistics” (NCHS) to evaluate the nutritional and health status of the US population.^[13]^ In this research, cross-sectional data were extracted from NHANES 2015 to 2018, encompassing a total of 19,225 individuals. Exclusion criteria were applied as follows: males (n = 9449), individuals with missing WWI data (n = 1699), those who did not complete the infertility questionnaire (n = 4708), and participants younger than 20 years or older than 50 years (n = 976). After applying these exclusions, 2393 eligible females were considered for the final analysis (Fig. 1).

### 2.3. WWI and BMI

The WWI was first suggested by Park et al in 2018.^[11]^ Subsequent studies have demonstrated that higher WWI values are positively linked with the occurrence of obesity.^[14]^ In NHANES, trained health experts measured participants’ WC and weight following standardized protocols. WWI was calculated as the ratio of WC (cm) to the square root of body weight (kg). In this study, both WWI and BMI were considered exposure variables. For the analyses, participants were grouped into Qs on the basis of their WWI and BMI distributions to facilitate subgroup comparisons.

### 2.4. Infertility

The outcome variable was defined utilizing self-reported information from the Reproductive Health Questionnaire (RQH074). Participants were asked, “Have you been trying for at least one year without getting pregnant?” A response of “yes” was classified as infertile, whereas a response of “no” was classified as fertile.^[8]^

### 2.5. Covariates

Covariates were also included that could influence the relationship between WWI and infertility. These variables were contraceptive pill use, history of pelvic infection/pelvic inflammatory disease (PID), regularity of menstrual periods over the last 12 months, age at menarche, smoking status, marital status, education level, race, family poverty-to-income ratio (PIR), and age. Participants were considered smokers if they had smoked 100 or more cigarettes in their lifetime.

### 2.6. Statistical analysis

Statistical analysis was conducted using EmpowerStats and R. This study followed the NHANES analysis guidelines, accounting for complex survey designs in all analyses, including sample weights (WTMEC2YR), masked variance pseudo-PSU (SDMVPSU), and masked variance pseudo-stratum (SDMVSTRA). For missing data in covariates, we used median imputation for continuous variables and mode imputation for categorical variables. *t*-tests and the χ^2^ test were used to assess differences in demographic and clinical characteristics among the 4 groups of participants. To further explore the respective associations between BMI and WWI with infertility, we employed multivariate logistic regression models. Three models were constructed through stepwise evaluation and adjustment for confounding factors. Model 1 included only the exposure variables (WWI and BMI) without adjusting for any covariates. Model 2 adjusted for age and race based on Model 1. Model 3 further adjusted for age, family PIR, race, education level, smoking status, marital status, age at menarche, history of pelvic infection/PID treatment, history of birth control pill use, and regular menstrual periods over the past 12 months based on Model 2. To evaluate dose-response relationships, WWI and BMI were categorized into Qs, and trend tests were performed. Additionally, we carried out subgroup analyses on the basis of age (<35/≥35 years), race, education level (<High school/High school/>High school), marital status, smoking status (yes/no), age at menarche (<10/10–14/≥15 years), regular menstruation in the past 12 months (yes/no), history of pelvic infection/PID treatment (yes/no), and history of birth control pill use (yes/no). The purpose of these analyses was to examine the association between WWI, BMI, and infertility across different sociodemographic groups. Interaction analyses assessed whether associations were consistent across subgroups. Additionally, smoothed curve fitting was utilized to examine potential nonlinear associations between BMI, WWI, and infertility. Piecewise linear regression models were used to further explore the threshold effect of BMI on infertility. The receiver operating characteristic (ROC) curve was drawn, and the area under the curve (AUC) was calculated to evaluate the predictive ability of BMI and WWI for infertility. Statistical significance was denoted as a two-sided *P*-value < .05.

## 3. Results

### 3.1. Baseline characteristics

Among the 2393 participants aged 20 to 50 years, 251 (10.49%) were identified as infertile. The mean age of participants was 35.06 ± 8.82 years, the mean WWI value was 11.03 ± 0.82, and the mean BMI value was 30.12 ± 8.29.

The study population’s baseline characteristics are shown in Table 1. Participants were categorized into 4 groups based on the Qs of the WWI: Q1 (lowest quartile) (<10.43), Q2 (10.43–10.98), Q3 (10.99–11.59), and Q4 (11.60–13.62), with roughly equal numbers in each group. Relative to participants in Q1, those in higher WWI tended to be older, had higher BMI values, tended to be Mexican American, and more frequently reported smoking. Furthermore, participants with lower family PIR scores and lower educational levels were more frequently represented in the higher WWI groups. The survey also showed that infertility was more common in the higher WWI groups (Q3 and Q4).

**Table 1:** Participant baseline characteristics according to WWI among US adults.

Characteristics	WWI				*P*-value
	Q1 (N = 598)	Q2 (N = 598)	Q3 (N = 598)	Q4 (N = 599)	
Age (yr)	32.25 (31.09,33.41)	35.02 (33.95, 36.08)	35.86 (34.92, 36.81)	36.49 (35.77, 37.21)	.0001
BMI (kg/m^2^)	24.34 (23.58, 25.10)	27.70 (27.07, 28.33)	31.67 (30.88, 32.45)	36.64 (35.52, 37.75)	<.0001
Family PIR	3.09 (2.89, 3.30)	2.83 (2.64, 3.02)	2.62 (2.43, 2.80)	2.37 (2.21, 2.53)	<.0001
Race (%)					
Mexican American	5.26 (2.90, 9.36)	8.75 (6.07, 12.46)	16.18 (11.43, 22.41)	16.22 (11.77, 21.92)	<.0001
Other Hispanic	6.68 (4.85, 9.12)	7.62 (5.28, 10.88)	9.36 (6.40, 13.50)	7.02 (5.01, 9.75)	
Non-Hispanic White	63.89 (56.98, 70.26)	59.14 (51.26, 66.58)	47.47 (41.04, 53.98)	55.35 (48.16, 62.32)	
Non-Hispanic Black	14.29 (10.83, 18.61)	11.70 (8.60, 15.72)	13.38 (9.84, 17.93)	12.83 (8.84, 18.26)	
Other races – including multi-racial	9.89 (7.65, 12.68)	12.79 (9.63, 16.80)	13.61 (10.58, 17.34)	8.59 (6.19, 11.80)	
Education level (%)					
<High school	5.53 (3.71, 8.16)	6.71 (4.78, 9.34)	13.65 (10.63, 17.36)	18.06 (14.67, 22.02)	<.0001
High school	15.65 (11.58, 20.83)	20.95 (15.71, 27.36)	20.32 (16.25, 25.11)	23.32 (19.88, 27.16)	
>High school	78.82 (72.77, 83.82)	72.34 (65.31, 78.42)	66.03 (60.12, 71.48)	58.62 (53.73, 63.34)	
Marital status (%)					
Married or living with a partner	54.97 (48.80, 60.99)	63.72 (58.74, 68.43)	64.09 (58.82, 69.04)	62.06 (57.13, 66.75)	.0205
Living alone	45.03 (39.01, 51.20)	36.28 (31.57, 41.26)	35.91 (30.96, 41.18)	37.94 (33.25, 42.87)	
Smoking (%)					
Yes	26.68 (22.18, 31.71)	32.82 (27.29, 38.87)	36.46 (32.25, 40.90)	38.39 (32.55, 44.58)	.0003
No	73.32 (68.29, 77.82)	67.18 (61.13, 72.71)	63.54 (59.10, 67.75)	61.61 (55.42, 67.45)	
Age when first menstrual period occurred (%)					
Age < 10	3.16 (1.71, 5.77)	3.07 (1.82, 5.13)	4.09 (2.49, 6.63)	4.24 (2.84, 6.30)	.0384
10 ≤ Age < 15	86.92 (82.82, 90.16)	90.93 (87.32, 93.58)	90.50 (87.02, 93.12)	91.59 (88.44, 93.94)	
15 ≤ Age	9.92 (7.04, 13.80)	6.01 (3.66, 9.69)	5.41 (3.21, 8.97)	4.16 (2.55, 6.73)	
Had regular periods in the past 12 mo (%)					
Yes	90.95 (87.77, 93.37)	83.13 (77.92, 87.32)	81.14 (75.98, 85.41)	81.45 (78.17, 84.34)	.0003
No	9.05 (6.63, 12.23)	16.87 (12.68, 22.08)	18.86 (14.59, 24.02)	18.55 (15.66, 21.83)	
Ever treated for a pelvic infection/PID (%)					
Yes	77.73 (72.63, 82.12)	77.36 (72.54, 81.55)	72.85 (67.23, 77.82)	72.71 (68.20, 76.81)	.1180
No	22.27 (17.88, 27.37)	22.64 (18.45, 27.46)	27.15 (22.18, 32.77)	27.29 (23.19, 31.80)	
Ever taken birth control pills (%)					
Yes	2.38 (1.60, 3.53)	5.61 (3.48, 8.93)	7.13 (3.97, 12.48)	6.02 (3.74, 9.55)	.0190
No	97.62 (96.47, 98.40)	94.39 (91.07, 96.52)	92.87 (87.52, 96.03)	93.98 (90.45, 96.26)	
Infertility (%)					
Yes	5.08 (3.42, 7.48)	12.44 (9.41, 16.28)	12.14 (9.68, 15.13)	16.04 (12.85, 19.83)	<.0001
No	94.92 (92.52, 96.58)	87.56 (83.72, 90.59)	87.86 (84.87, 90.32)	83.96 (80.17, 87.15)	

Note: For continuous variables: survey-weighted mean (95% CI); *P*-values were calculated using survey-weighted linear regression (svyglm). For categorical variables: survey-weighted percentages (95% CI); *P*-values were obtained via survey-weighted chi-square test (svytable).

BMI = body mass index, CI = confidence interval, PID = pelvic inflammatory disease, PIR = poverty-to-income ratio, WWI = weight-adjusted waist index.

Note: For continuous variables: survey-weighted mean (95% CI); *P*-values were calculated using survey-weighted linear regression (svyglm). For categorical variables: survey-weighted percentages (95% CI); *P*-values were obtained via survey-weighted chi-square test (svytable).

### 3.2. Link between WWI, BMI, and infertility

The link between infertility and WWI is summarized in Table 2. A significant positive correlation was observed across all models. In Model 1 (unadjusted), higher WWI was linked with raised infertility odds (OR = 1.61, 95% CI: 1.39–1.85). This association remained consistent after adjusting for age and race (Model 2: OR = 1.57, 95% CI: 1.35–1.83) and in the fully adjusted model (Model 3: OR = 1.61, 95% CI: 1.34–1.95). Following full adjustment, each 1-unit rise in WWI was linked to a 61% higher infertility’s odds. For further analysis, WWI was divided into Qs. Women in the highest quartile (Q4) exhibited significantly higher odds of infertility compared to those in the lowest quartile (Q1), indicating that the likelihood of infertility increases with rising WWI. This association remained statistically significant in Model 3 (OR = 3.63, 95% CI: 1.96–6.73).

**Table 2:** The associations between WWI and infertility.

Exposure	Model 1 [OR (95% CI)]	Model 2 [OR (95% CI)]	Model 3 [OR (95% CI)]
WWI (continuous)	1.61 (1.39, 1.85)	1.57 (1.35, 1.83)	1.61 (1.34, 1.95)
WWI (quartile)			
Quartile 1	Reference	Reference	Reference
Quartile 2	2.66 (1.62, 4.37)	2.56 (4.53, 4.30)	2.58 (1.44, 4.61)
Quartile 3	2.58 (1.60, 4.19)	2.57 (1.54, 4.27)	2.57 (1.40, 4.70)
Quartile 4	3.57 (2.17, 5.89)	3.41 (2.01, 5.76)	3.63 (1.96, 6.73)
*P* for trend	<.0001	.0001	.0003

Note: Model 1: No adjustment of covariates; Model 2: Adjusted by race and age; Model 3: Adjusted by age, family size, race, marital status, age when 1st menstrual period occurred, smoking, education level, ever treated for a pelvic infection/PID, ever used birth control pills, regular periods over the last 12 mo.

CI = confidence interval, OR = odds ratio, PID = pelvic inflammatory disease, WWI = weight-adjusted waist index.

Table 3 shows the link between BMI and infertility. The results indicate that in Model 1 (OR = 1.04, 95% CI: 1.02–1.05) and Model 2 (OR = 1.03, 95% CI: 1.01–1.06), BMI was positively correlated with infertility. In Model 3 (fully adjusted model), this link remained significant, although it was less pronounced than that of WWI (OR = 1.04, 95% CI: 1.02–1.06). Participants were divided into 4 groups based on BMI Qs: Q1 (<23.5), Q2 (23.6–28.6), Q3 (28.7–34.7), and Q4 (34.8–66.2). Compared with Q1, participants in Q4 showed a 127% increased risk of infertility (OR = 2.27, 95% CI: 1.29–4.01).

**Table 3:** The associations between BMI and infertility.

Exposure	Model 1 [OR (95% CI)]	Model 2 [OR (95% CI)]	Model 3 [OR (95% CI)]
BMI (continuous)	1.04 (1.02, 1.05)	1.03 (1.01, 1.06)	1.04 (1.02, 1.06)
BMI (quartile)			
Quartile 1	Reference	Reference	Reference
Quartile 2	0.95 (0.51, 1.75)	0.90 (0.48, 1.71)	0.92 (0.49, 1.74)
Quartile 3	1.27 (0.71, 2.27)	1.23 (0.68, 2.24)	1.23 (0.66, 2.30)
Quartile 4	2.28 (1.41, 3.69)	2.23 (1.29, 3.85)	2.27 (1.29, 4.01)
*P* for trend	.0006	.0023	.0045

Note: Model 1: No adjustment of covariates; Model 2: Adjusted by race and age; Model 3: Adjusted by age, family size, race, marital status, age when 1st menstrual period occurred, smoking, education level, ever treated for a pelvic infection/PID, ever used birth control pills, regular periods over the last 12 mo.

BMI = body mass index, CI = confidence interval, OR = odds ratio, PID = pelvic inflammatory disease.

**Figure 2.**
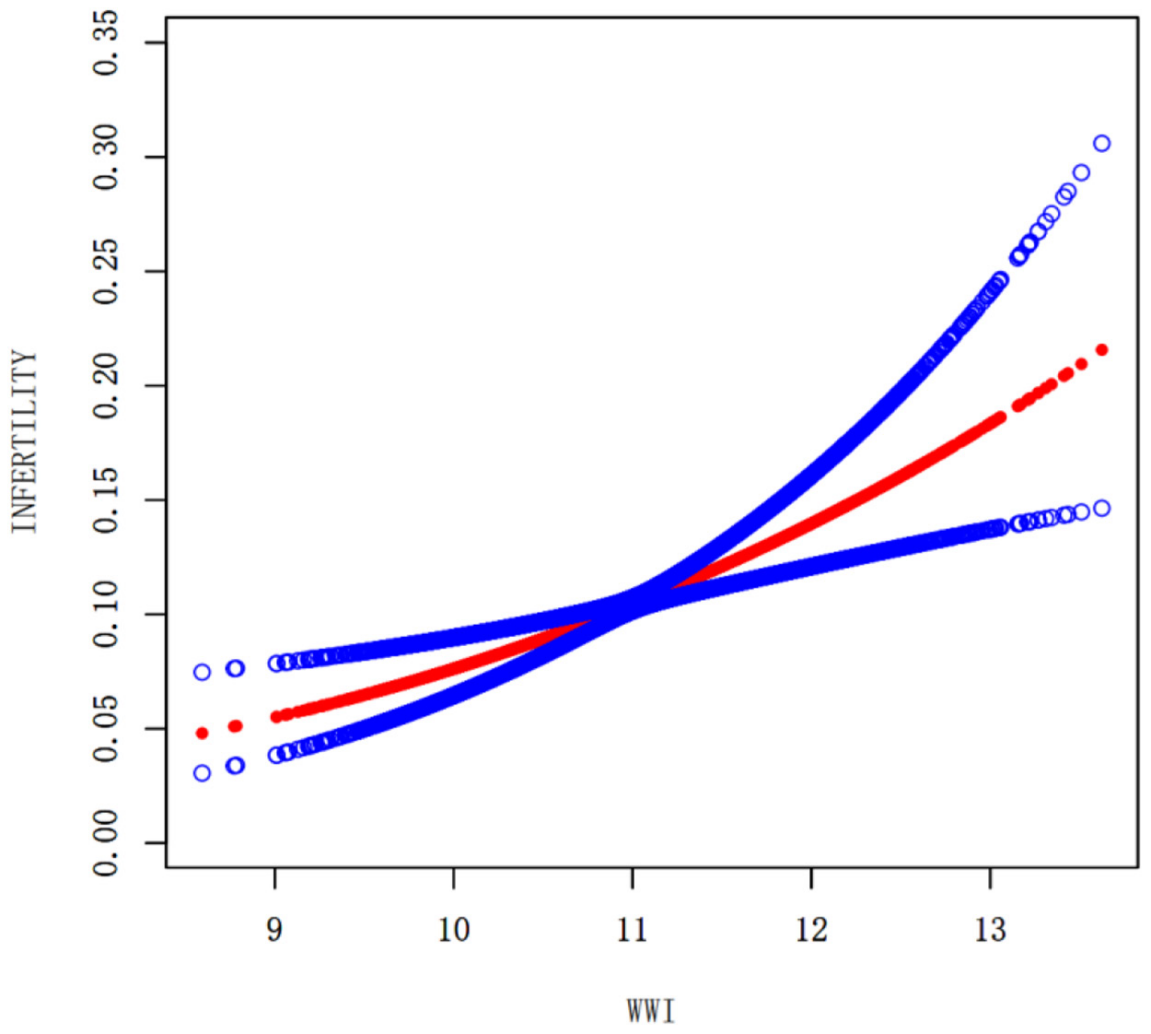
Smooth curve fitting for WWI and infertility. WWI = weight-adjusted waist index.

**Figure 3.**
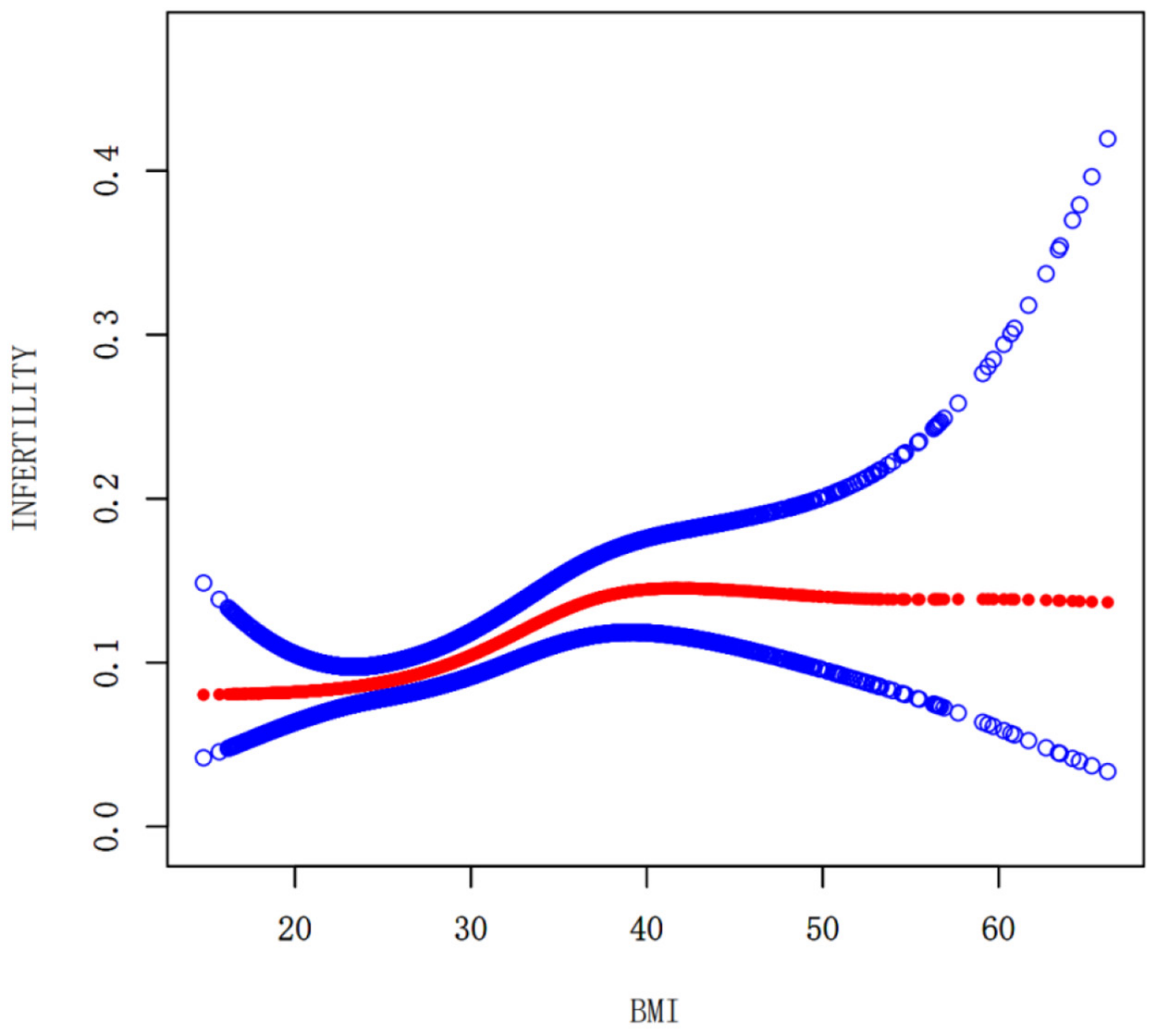
Smooth curve fitting for BMI and infertility. BMI = body mass index.

Additionally, curve-fitting analysis revealed nonlinear relationships between WWI and infertility, as well as between BMI and infertility (Figs. 2 and 3). WWI showed a monotonically increasing trend with infertility risk overall, meaning higher WWI levels corresponded to greater infertility risk. The risk of infertility associated with BMI was slightly elevated at low BMI levels, then increased sharply as BMI rose. Therefore, we further calculated the potential inflection point (K) in the association between BMI and infertility using threshold effect analysis. Results showed that in Model 1 (standard linear model), regardless of covariate adjustment, each 1-unit increase in BMI was associated with approximately a 3% increase in infertility risk, with statistically significant results (*P* < .05). The inflection points in Model 2 were 36.6 and 19.8 before and after adjustment, respectively. BMI exhibited a bidirectional association with infertility: on the left side (BMI < 19.8), it was negatively correlated with infertility (OR = 0.71, 95% CI: 0.52–0.98, *P* for trend = .0378), while the right side (BMI ≥ 19.8) showed a positive correlation with infertility (OR = 1.03, 95% CI: 1.01–1.05, *P* for trend = .0005). Before and after covariate adjustment, the log-likelihood ratio test *P* values were .083 and .043, respectively (Table 4).

**Table 4:** Threshold effect of BMI on infertility.

	Before adjustment	After adjustment
Model 1		
OR (95% CI)	1.03 (1.01, 1.04)	1.03 (1.01, 1.04)
*P* for trend	.0003	.0022
Model 2		
Breakpoint (K)	36.6	19.8
OR1 (< K)	1.05 (1.02, 1.07)	0.71 (0.52, 0.98)
	.0005	.0378
OR2 (> K)	1.00 (0.96, 1.04)	1.03 (1.01, 1.05)
	.9521	.0005
OR2/OR1	0.95 (0.90, 1.01)	1.44 (1.04, 2.00)
	.0887	.0261
Logarithmic likelihood ratio test *P* value	.083	.043

Note: Model 1: Standard linear model; Model 2: Two-piecewise linear model; Before adjustment: Covariates were not adjusted at all. After adjustment: Adjusted for age, family size, race/ethnicity, education level, marital status, smoking, age when first menstrual period occurred, ever treated for a pelvic infection/PID, ever taken birth control pills, had regular periods in the past 12 mo.

BMI = body mass index, CI = confidence interval, OR = odds ratio, PID = pelvic inflammatory disease.

### 3.3. Subgroup analyses

We conducted interaction tests and stratified subgroup analyses on the basis of age, race, education level, smoking status, age at menarche, marital status, menstrual regularity over the past 12 months, history of pelvic infection/PID treatment, and birth control pill use’s history to evaluate whether the associations between WWI, BMI, and infertility were consistent across different population subgroups (Table 5).

**Table 5:** Subgroup analysis of the link between WWI and BMI and infertility.

Subgroup	WWI		BMI	
	Infertility [OR (95% CI)]	*P* for interaction	Infertility [OR (95% CI)]	*P* for interaction
Age				
<35 yr	2.13 (1.75, 2.59)	.0016	1.05 (1.02, 1.07)	.1784
≥35 yr	1.32 (1.03, 1.68)		1.03 (1.00, 1.05)	
Race				
Mexican American	1.29 (0.88, 1.88)	.0087	1.06 (1.01, 1.11)	.1212
Other Hispanic	1.39 (0.73, 2.62)		1.05 (0.98, 1.12)	
Non-Hispanic White	1.93 (1.61, 2.31)		1.04 (1.02, 1.07)	
Non-Hispanic Black	1.06 (0.76, 1.49)		1.00 (0.97, 1.04)	
Other races – including multi-racial	1.06 (0.61, 1.85)		1.01 (0.97, 1.06)	
Education level				
<High school	1.78 (1.13, 2.81)	.0466	1.09 (1.04, 1.14)	.1091
High school	1.78 (1.13, 2.81)		1.02 (1.00, 1.05)	
>High school	1.78 (1.39, 2.28)		1.03 (1.00, 1.06)	
Marital status				
Married or living with a partner	1.56 (1.26, 1.93)	.5261	1.04 (1.01, 1.06)	.7181
Living alone	1.71 (1.37, 2.13)		1.03 (1.00, 1.06)	
Smoking				
Yes	1.58 (1.24, 2.02)	.8799	1.03 (1.01, 1.06)	.6353
No	1.62 (1.29, 2.05)		1.04 (1.01, 1.07)	
Age when first menstrual period occurred				
Age < 10	1.15 (0.68, 1.96)	.3314	1.00 (0.94, 1.07)	.1042
10 ≤ Age < 15	1.62 (1.35, 1.95)		1.03 (1.01, 1.06)	
≥15	1.88 (1.16, 3.07)		1.07 (1.02, 1.12)	
Had regular periods in the past 12 mo				
Yes	1.64 (1.38, 1.95)	.6591	1.04 (1.01, 1.06)	.9944
No	1.43 (0.79, 2.59)		1.04 (0.97, 1.10)	
Ever treated for a pelvic infection/PID				
Yes	1.08 (0.63, 1.87)	.1584	1.01 (0.93, 1.10)	.5347
No	1.66 (1.39, 1.98)		1.04 (1.02, 1.06)	
Ever taken birth control pills				
Yes	1.73 (1.43, 2.11)	.1697	1.03 (1.01, 1.05)	.3259
No	1.24 (0.81, 1.88)		1.05 (1.02, 1.08)	

Note: Subgroup analysis of the link between infertility and WWI showed that none of the stratifications, such as marital status, smoking, age at menarche, history of pelvic infection/PID treatment, regular menstruation in the past 12 mo, and history of contraceptive use, significantly altered the positive association between WWI and infertility.Subgroup analysis of the link between BMI and infertility indicated that none of the stratifications significantly modified the positive link between BMI and infertility.

BMI = body mass index, CI = confidence interval, OR = odds ratio, PID = pelvic inflammatory disease, WWI = weight-adjusted waist index.

In the subgroup analysis of WWI, significant associations with infertility were observed in the subgroups of education level, age, and race (*P* < .05). No significant associations were found in the subgroups of marital status, smoking history, age at menarche, menstrual regularity over the last 12 months, pelvic infection/PID treatment history, and contraceptive use history (*P* > .05). In contrast, the subgroup analysis of BMI revealed no significant effect modifiers.

### 3.4. ROC curve for infertility

**Figure 4.**
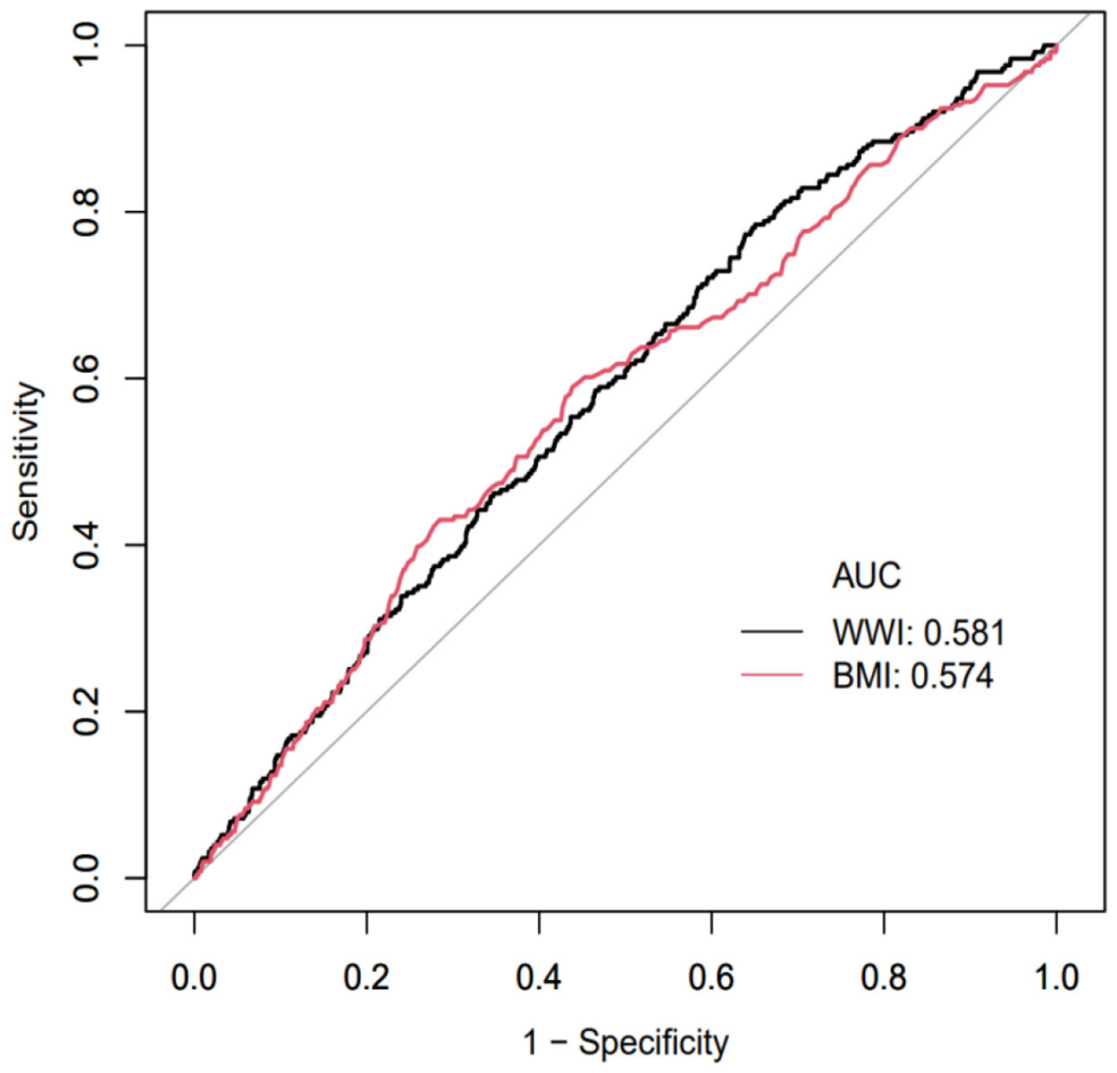
ROC curve for infertility. ROC = receiver operating characteristic.

We utilized ROC curve analysis to estimate the diagnostic performance of WWI and BMI for infertility. The results showed that the AUC for WWI was 0.581, while the AUC for BMI was 0.574 (Fig. 4).

## 4. Discussion

This study scrutinized the link between WWI, BMI, and infertility among U.S. women aged 20 to 50 years using data from the 2015 to 2018 NHANES. Among 2395 participants, we observed a substantial positive link between both WWI and BMI with infertility, implying that women with higher WWI or BMI were more likely to experience infertility. Moreover, subgroup analyses illustrated that the link between WWI and infertility varied by age and race but remained consistent across other demographic characteristics. By contrast, the link between BMI and infertility was stable across all subgroups. Smooth curve fitting further confirmed a nonlinear link between infertility and both WWI and BMI. The AUC values for BMI and WWI were 0.574 and 0.581, respectively.

Most studies still use BMI and WC to judge obesity; however, BMI, which is based solely on body weight, may not fully capture an individual’s obesity status.^[15]^ Evidence suggests that women with central (centripetal) obesity are less likely to conceive in each cycle.^[16]^ The WWI (cm/√kg) provides a more accurate characterization of both the degree and type of obesity. As a novel indicator, WWI has been applied in several research fields to assess obesity. Consistent with these findings, our study demonstrated that infertility in women was positively linked with both WWI (OR = 3.63, 95% CI: 1.96–6.73) and BMI (OR = 2.27, 95% CI: 1.29–4.01), with WWI showing a stronger correlation than BMI.

The results indicate that obesity is significantly and positively linked with the occurrence of infertility. Following adjustment for all covariates, each unit rise in WWI was linked with 61% higher infertility’s odds, while each unit rise in BMI was linked with 4% higher odds. Previous studies have similarly demonstrated that obesity contributes to infertility.^[7]^ The results of a systematic review and meta-analysis showed that overweight/obesity (FOR = 0.85; 95% CI: 0.80–0.90) was associated with significantly prolonged time to pregnancy (reduced fertility) and increased risk of infertility (OR = 1.60, 95% CI: 1.31–1.94).^[17]^ A prospective cohort study from the Netherlands reported that for each unit increase in BMI in both sexes, the ability to conceive decreased (women: FR = 0.98, 95% CI: 0.97–0.99; male: FR = 0.99, 95% CI: 0.98–1.00), and higher BMI was associated with infertility (female: OR = 1.04, 95% CI: 1.02–1.05; male: OR = 1.03, 95% CI: 1.00–1.06).^[18]^ Similarly, a prospective cohort study among Black women showed that, after adjusting for BMI, a larger WC (>84 cm) or waist-to-hip ratio (WHR > 0.85) was significantly associated with reduced fertility.^[19]^ Collectively, these findings reinforce the association between obesity and infertility, consistent with the results of our study.

Additionally, this study found a nonlinear, two-stage relationship between BMI and female infertility, with an inflection point around 19.8 kg/m^2^. Below this threshold, the risk of infertility decreases as BMI increases; above it, the risk gradually rises with increasing BMI. This suggests that both underweight and overweight conditions may adversely affect female reproductive health. This nonlinear pattern aligns with findings from multiple recent studies. Hernáez et al observed that both higher and lower BMIs were associated with increased risks of reduced fertility.^[20]^ Another study from China demonstrated that underweight, overweight, or obese status in women, as well as male status, was linked to prolonged time to pregnancy, suggesting that a moderate weight level is most conducive to fertility.^[21]^

Although a correlation between obesity and infertility has been established, the underlying mechanisms remain incompletely understood. Gonnella F et al reported that obesity and excess adiposity in women can influence endometrial gene expression and immune tolerance, directly impacting the hypothalamic-pituitary-ovarian axis, ovarian function, oocyte quality, and the endometrium, thereby impairing fertility.^[22]^ Abnormal secretion of metabolic hormones is associated with altered ovarian function and ovulation regulation. For example, obesity is often accompanied by insulin resistance and compensatory hyperinsulinemia. Excessive insulin levels can directly affect the ovary by interfering with gonadotropin signaling and abnormally amplifying androgen synthesis in theca cells to affect reproductive function.^[23]^ In addition, adipose tissue is an active endocrine organ. In the context of obesity, the abnormal changes in adipokines such as leptin and adiponectin secreted by adipose tissue lead to the disorder of the hormonal environment, which may affect the endometrial environment and make it less sensitive to embryo implantation and early pregnancy.^[24]^ In addition, obese women are more likely to experience pathological conditions such as polycystic ovary syndrome and menstrual irregularities,^[25,26]^ both of which further contribute to ovulatory dysfunction. Persistent energy deficiency (caused by inadequate diet, low body weight, or intense exercise) suppresses neuroendocrine signaling in the hypothalamic-pituitary-gonadal axis, leading to menstrual dysfunction, low estrogen levels, and anovulation.^[27]^ Obesity and central adiposity can induce chronic inflammation and elevated oxidative stress levels, adversely affecting embryo implantation and early embryonic development^[28,29]^ Beyond biological mechanisms, the psychosocial burden of infertility itself may promote weight gain, creating a feedback loop that aggravates obesity and further compromises reproductive outcomes. This study has certain limitations. Firstly, due to the cross-sectional design of this study, a causal relationship between WWI, BMI, and infertility could not be confirmed. Second, although we adjusted for multiple confounding factors, we cannot completely rule out the effect of residual or unmeasured confounding factors, such as lifestyle factors, psychological factors, and genetic diseases, that may have affected the relationship between obesity measures and infertility. Furthermore, due to database limitations, some variables related to female infertility were not available, which may have limited the comprehensiveness of our analysis. In addition, measurement errors in exposure factors or self-reported errors in outcome factors may lead to information bias, and the processing of missing data may aggravate the selection bias, which may affect the accuracy of effect estimation. Notwithstanding these limitations, this study also has important strengths. The study utilized a nationally representative large sample, which strengthens the generalizability of the results. Adjustments were made for a broad set of covariates to improve the reliability of the results. Moreover, subgroup analyses were carried out to estimate the uniformity of the link between infertility and WWI across several populations. Finally, by directly comparing traditional obesity indicators (such as BMI) with an emerging measure (WWI), this study provides novel evidence on the relative utility of these metrics in infertility research.

## 5. Conclusion

In summary, our study demonstrated that both WWI and BMI are positively associated with the incidence of infertility, suggesting that these measures may have potential value in identifying women at risk of infertility in clinical settings. These findings highlight the need for future research to identify more discriminative biomarkers or to develop multi-indicator predictive models that integrate WWI, BMI, and other clinical or biochemical parameters, thereby improving the accuracy and reliability of infertility diagnosis.

## Acknowledgments

A special thanks to all of the NHANES participants who freely gave their time to make this and other studies possible.

## Author contributions

**Conceptualization:** Siqi Zhang, Xiaoming Huang.

**Data curation:** Siqi Zhang, Xiaoming Huang.

**Formal analysis:** Siqi Zhang, Yulu Zhou, Xiaoming Huang.

**Funding acquisition:** Siqi Zhang, Yulu Zhou, Xiaoming Huang.

**Investigation:** Siqi Zhang, Yulu Zhou, Xiaoming Huang.

**Methodology:** Siqi Zhang, Junjie Cao, Xiaoming Huang.

**Project administration:** Siqi Zhang, Yulu Zhou, Yixuan He, Junjie Cao, Xiaoming Huang.

**Resources:** Siqi Zhang, Yixuan He, Junjie Cao, Xiaoming Huang.

**Software:** Siqi Zhang, Junjie Cao, Xiaoming Huang.

**Supervision:** Siqi Zhang, Baoyi Liao, Yixuan He, Xiaoming Huang.

**Validation:** Siqi Zhang, Baoyi Liao, Yixuan He, Xiaoming Huang.

**Visualization:** Siqi Zhang, Baoyi Liao, Xiaoming Huang.

**Writing – original draft:** Siqi Zhang, Baoyi Liao, Xiaoming Huang.

**Writing – review & editing:** Siqi Zhang, Baoyi Liao, Xiaoming Huang.
